# Supplementary material for: HDfleX: Software for Flexible High Structural Resolution of Hydrogen/Deuterium-Exchange Mass Spectrometry Data
Source: Anal Chem. 2022 Mar 9;94(11):4557–64. doi: 10.1021/acs.analchem.1c05339 (PMC9204700; doi:10.1021/acs.analchem.1c05339)
Supplement: Supplementary file 1 — ac1c05339_si_001.pdf [file ac1c05339_si_001.pdf]

Supporting information:

HDfleX: Software for flexible high structural resolution of hydrogen/deuterium-exchange mass spectrometry data

Neeleema Seetaloo[1], Monika Kish[1], Jonathan J. Phillips[1,2]*

[1]*Living Systems Institute, Department of Biosciences, University of Exeter, Stocker Road, Exeter, EX4 4QD, UK*

[2]*Alan Turing Institute, British Library, London, NW1 2DB, UK*

*Correspondence to: jj.phillips@exeter.ac.uk

SI Materials

All reagents and chemicals were purchased from Sigma-Aldrich, USA, unless mentioned otherwise.

SI Methods

Bradykinin HDX-MS experimental details

0.5 µL of 10 mg/mL bradykinin peptide in dimethyl sulfoxide was added to 500 µL of each of the equilibration buffers (Table S1: Buffers 1-4) and allowed to equilibrate at room temperature for 1 hour. HDX for timepoints under 300 s was performed using a fully automated, millisecond HDX labelling and online quench-flow instrument, ms2min (Applied Photophysics, UK). HDX for timepoints above 300 s were performed using a CTC PAL sample handling robot (LEAP Technologies, USA). The ms2min or CTC PAL robot was connected to an HDX manager (Waters, USA). Mass spectrometry (MS) data were acquired on a Waters Synapt G2-Si Q-IM-TOF instrument. Three technical replicates and seven timepoints were collected. For Buffers 1-3, the timepoints were: 50 ms, 100 ms, 250 ms, 500 ms, 5000 ms, 60 s, 300 s. For Buffer 4, the timepoints were: 10 s, 20 s, 50 s, 100 s, 998 s, 11972 s, 59858 s. Deuterium incorporation was determined in DynamX 3.0 (Waters, USA). Back-exchange correction was performed using the uptake plateau determination method in HDfleX.

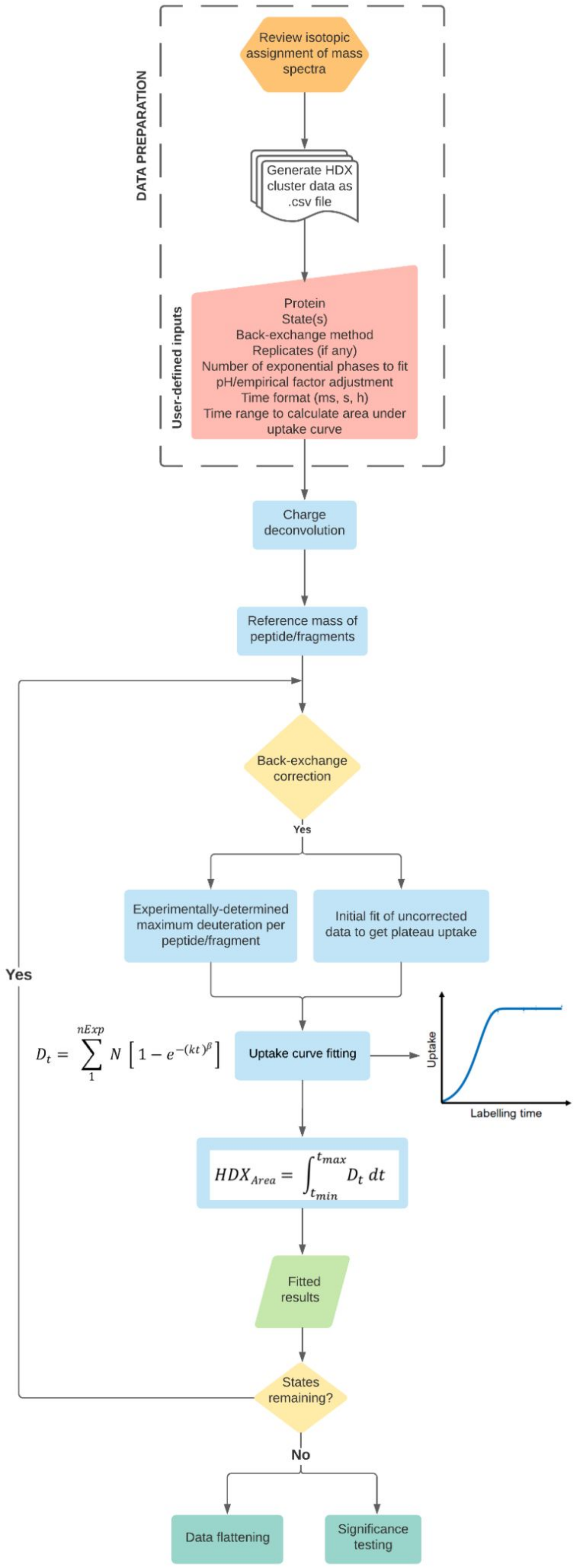

Figure S1: Flowchart describing the import and fitting stage of HDfleX

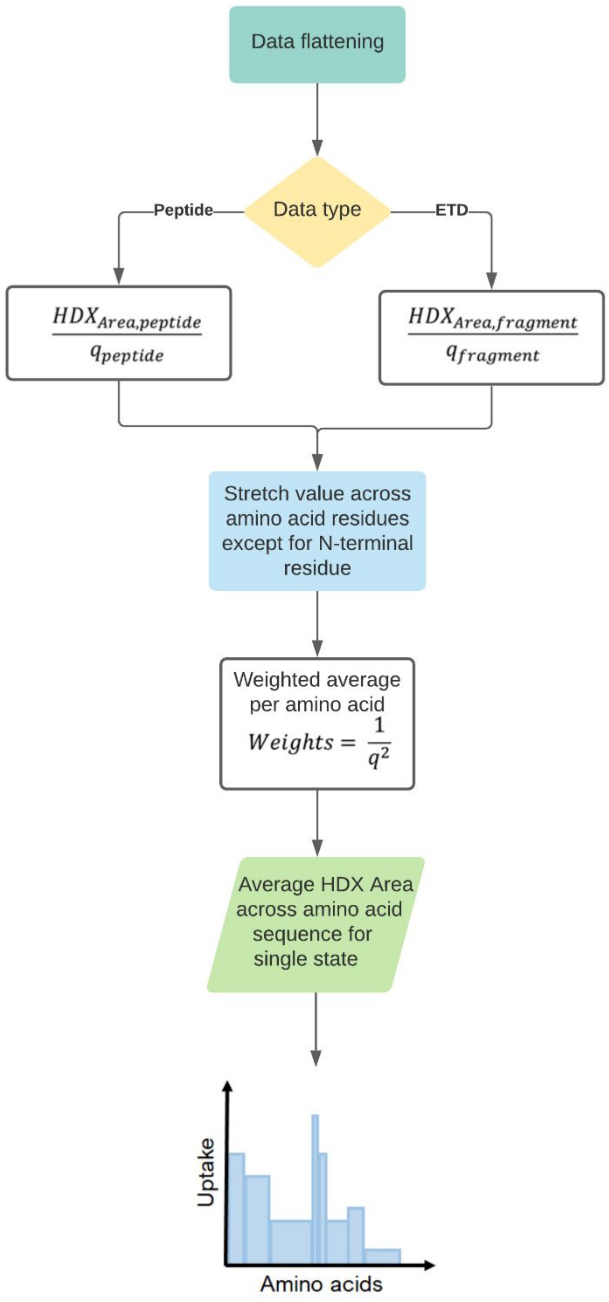

Figure S2: Flowchart describing the data flattening stage of HDfleX.

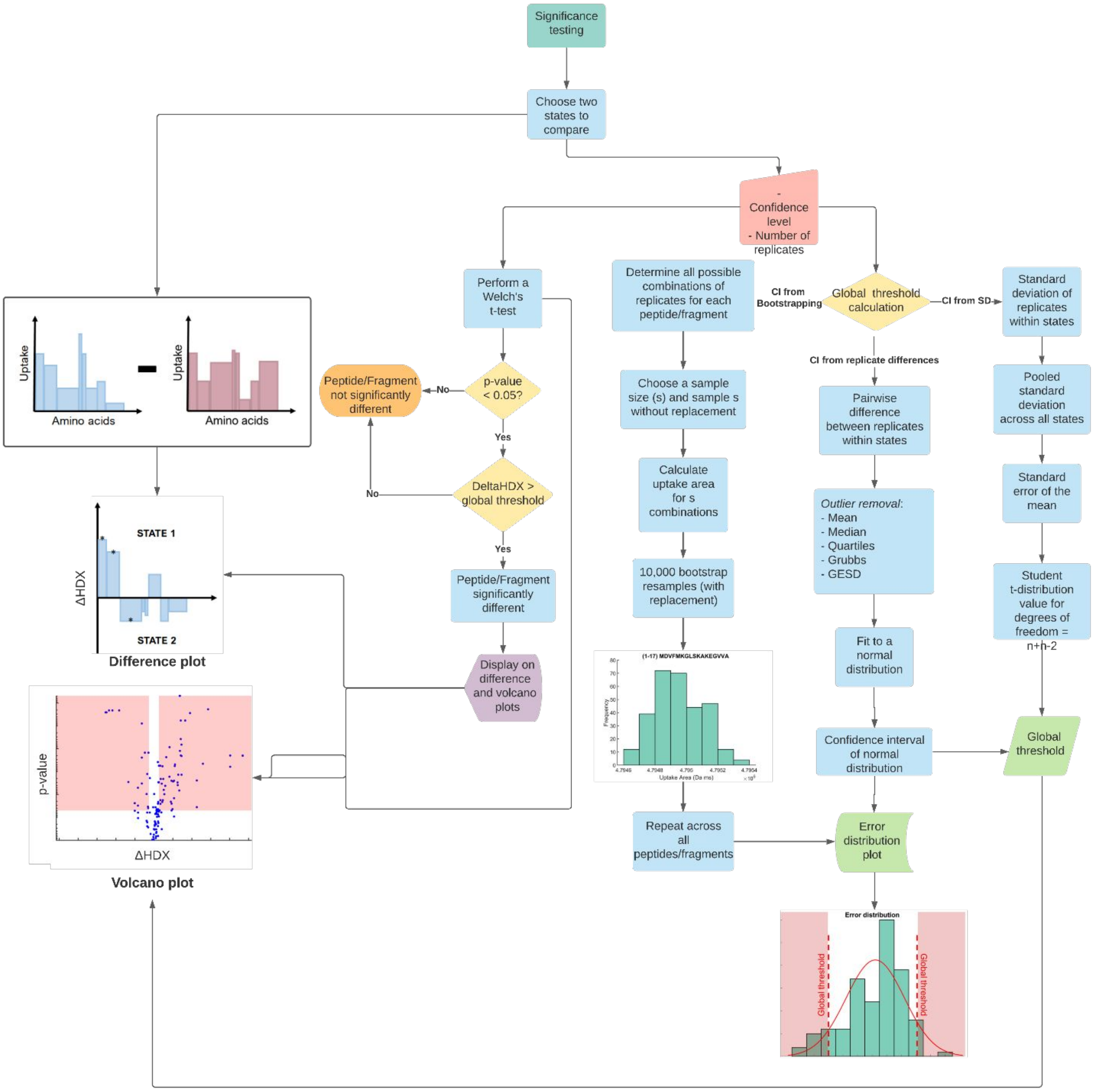

Figure S3: Flowchart describing the statistical significance analysis stage of HDfleX.

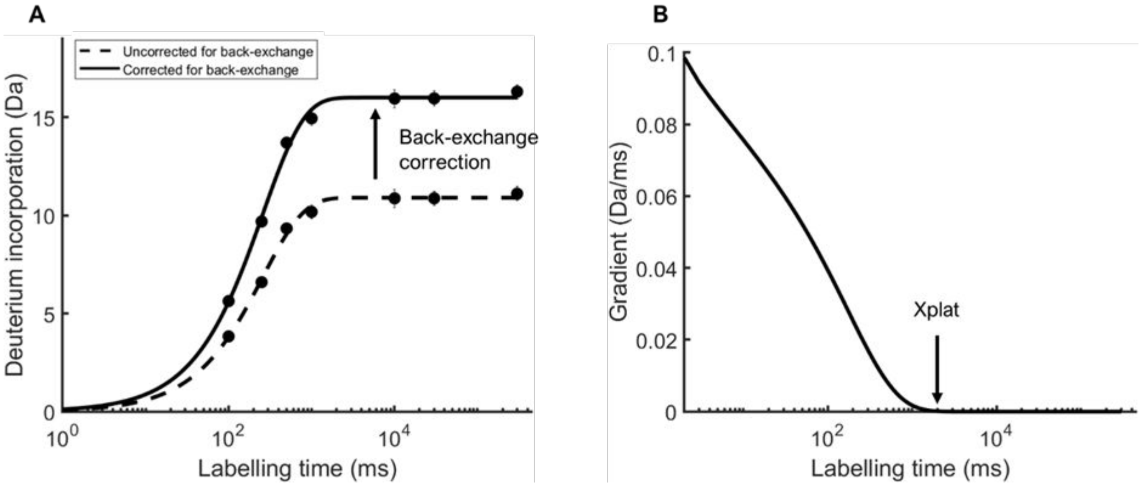

Figure S4: Plateau back-exchange determination. (A) Back-exchange correction shifts the uncorrected uptake curve (dotted) to the corrected curve (solid) for an example peptide; (B) The gradient of the fitted uncorrected curve is determined per time unit. The time at which the gradient is < 0.0001 is called Xplat and the uptake at Xplat is the maxD.

Table S1: Buffer compositions.

**Ion/Additive**	**Buffer 1 (mM)**	**Buffer 2 (mM)**	**Buffer 3 (mM)**	**Buffer 4 (mM)**	**Buffer 5 (mM)**
**Na^+**	0	143	15	20	0
**K^+**	0	4	140	60	0
**Ca^{2+}**	0	2.5	0.0001	0	0
**Mg^{2+}**	0	0.7	10	0	0
**TCEP**	0	0	0	0	1
**pH**	7.4	7.4	7.2	4.9	7.0
**Buffer system**	20 mM Tris	20 mM Tris	20 mM Tris	20 mM citrate	40 mM Tris

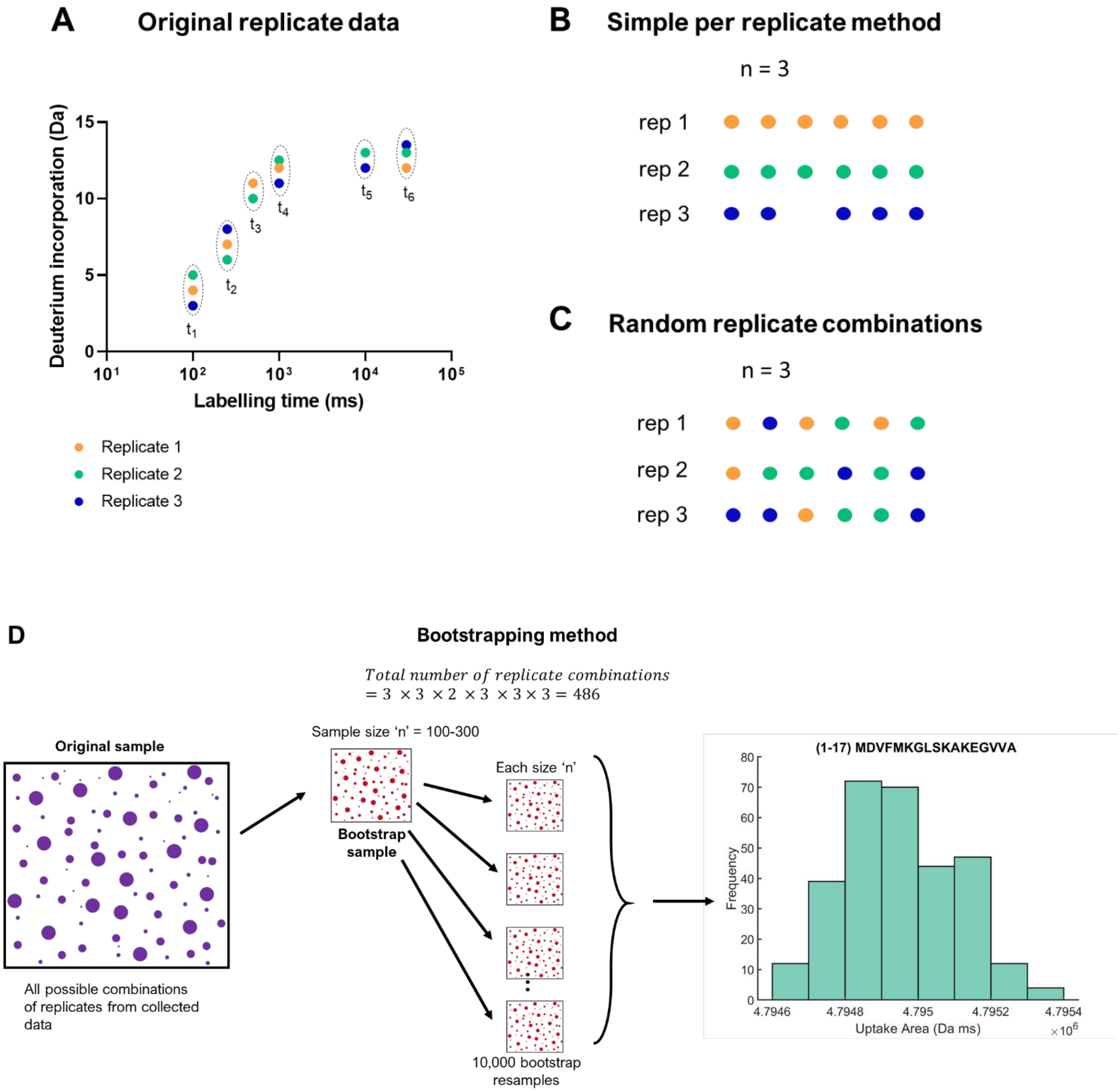

Figure S5: Methods of data distribution by separating the replicates; **(A)** Original replicate data for a chosen peptide with data points coloured as follows: replicate 1 = orange, replicate 2 = green, replicate 3 = blue. Each timepoint is encircled with broken lines; **(B)** Simple per replicate method: one replicate is chosen at each timepoint in numerical order until all experimental replicates have been processed; **(C)** Random replicate combinations method: A random replicate data point from the available replicates is chosen at each timepoint and this is repeated n times; **(D)** Bootstrapping method: All possible combinations of replicates at all timepoints are fitted and the uptake area is calculated. A bootstrap sample size of 300 was chosen here, and this sample size is resampled with replacement 10,000 times. The resulting uptake area distribution is shown for the chosen peptide, from which the mean and standard deviation can be determined.

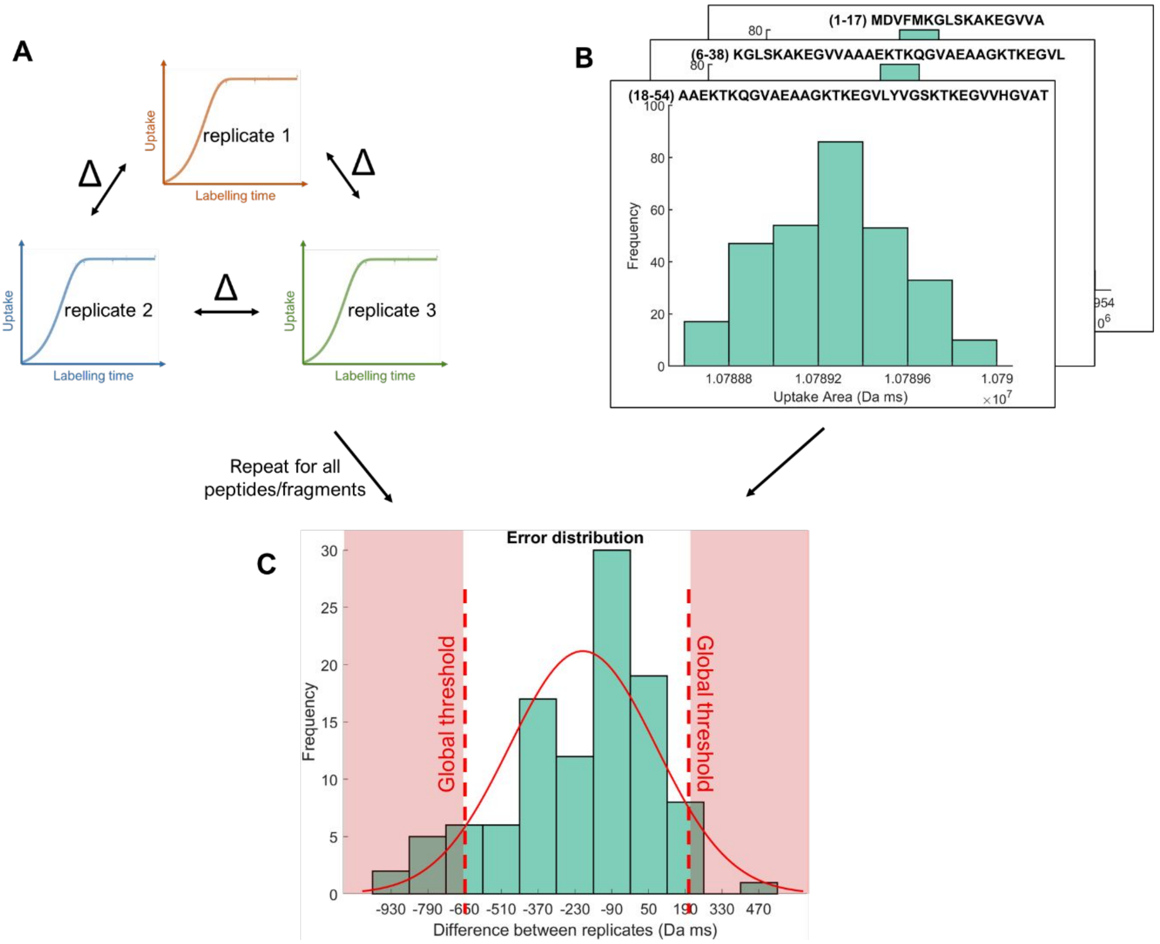

Figure S6: Error distribution of entire dataset using two different methods. (A) When the simple per replicate or random replicate combinations method of data distribution are used, the pairwise difference between the replicates for a peptide/fragment within each state is calculated. These pairwise differences are subsequently fitted with a normal distribution; (B) When using bootstrapping method of data distribution, the error distribution is determined from the maximum confidence interval of the mean across all peptides and fragments. (C) Resulting error distribution: the red shaded region represents differences that are large enough to be significant.

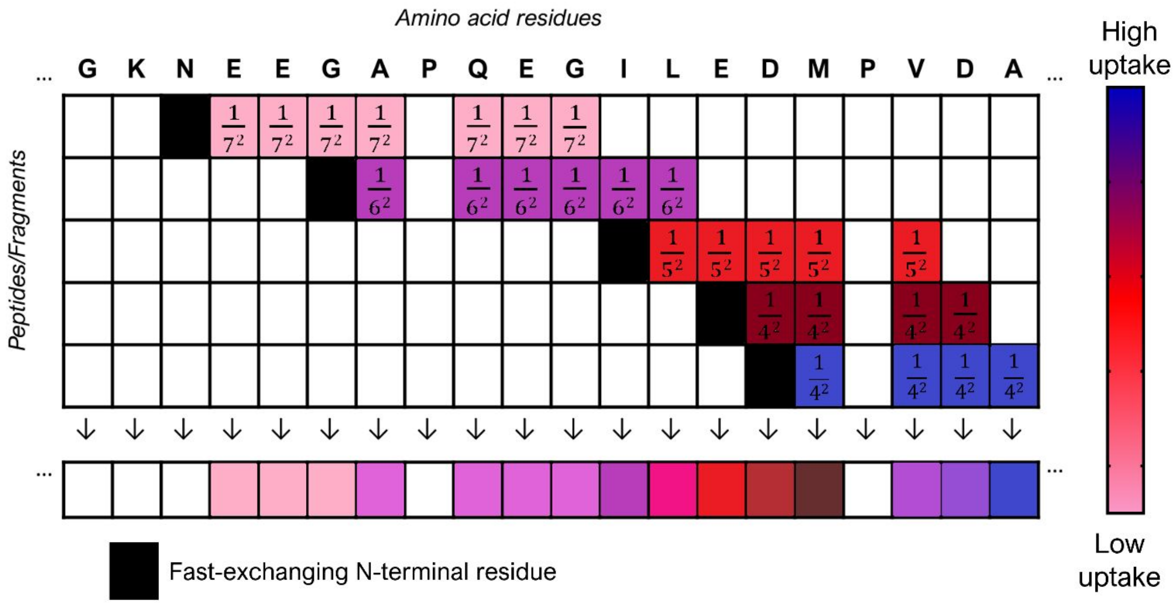

Figure S7: Schematic of the data flattening process adapted from Keppel & Weis, 2014[3]. The different colours represent different uptake/area values, with blanks representing no uptake/area, and the black squares representing rapidly exchanging N-terminal amide hydrogens. The bottom grid shows the flattened data which is obtained by calculating a weighted average of the uptake/area at each amino acid (per column) given by Equation 3.

Testing various weights for the peptides and fragments, including subtraction of consecutive ETD fragments

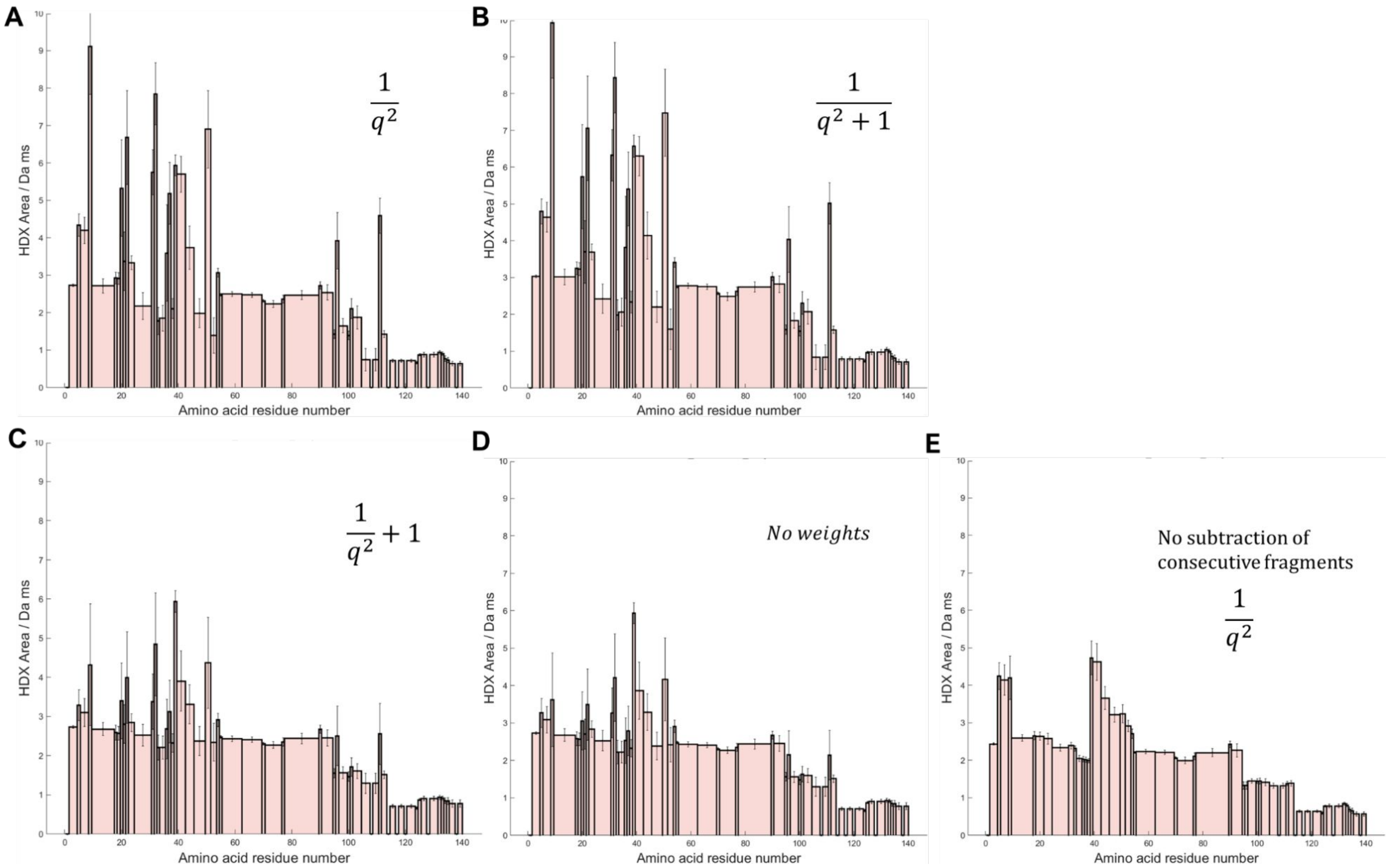

Figure S8: A range of weights were tested for the flattening of the peptides and ETD fragments; the weights are shown alongside the plots. In (A)-(C), the flattening was performed using the weights shown, as well as the subtraction of consecutive ETD fragments; (D) No weights were used but subtraction of consecutive ETD fragments was still performed; (E) Weight of 1/q^2 was used and no subtraction of the fragments performed.

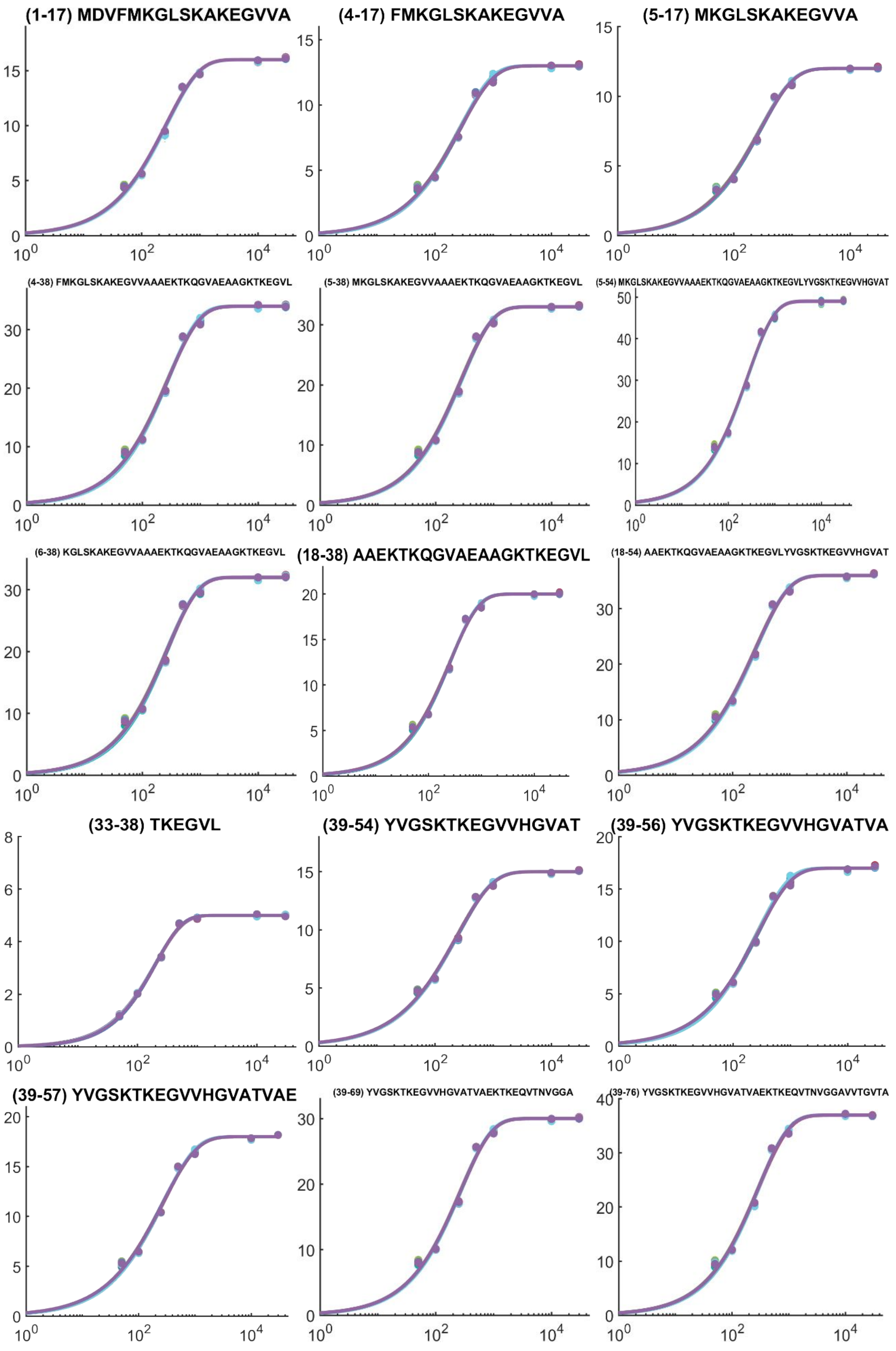

(1-17) MDVFMKGLSKAKEGVVA
(4-17) FMKGLSKAKEGVVA
(5-17) MKGLSKAKEGVVA
(4-38) FMKGLSKAKEGVVAAAEKTKQGVAEAAGKTKEGVL
(5-38) MKGLSKAKEGVVAAAEKTKQGVAEAAGKTKEGVL
(5-54) MKGLSKAKEGVVAAAEKTKQGVAEAAGKTKEGVLYVGSKTKEGVVHGVAT
(6-38) KGLSKAKEGVVAAAEKTKQGVAEAAGKTKEGVL
(18-38) AAEKTKQGVAEAAGKTKEGVL
(18-54) AAEKTKQGVAEAAGKTKEGVLYVGSKTKEGVVHGVAT
(33-38) TKEGVL
(39-54) YVGSKTKEGVVHGVAT
(39-56) YVGSKTKEGVVHGVATVA
(39-57) YVGSKTKEGVVHGVATVAE
(39-69) YVGSKTKEGVVHGVATVAEKTKEQVTNVGGA
(39-76) YVGSKTKEGVVHGVATVAEKTKEQVTNVGGAVVTGVTA

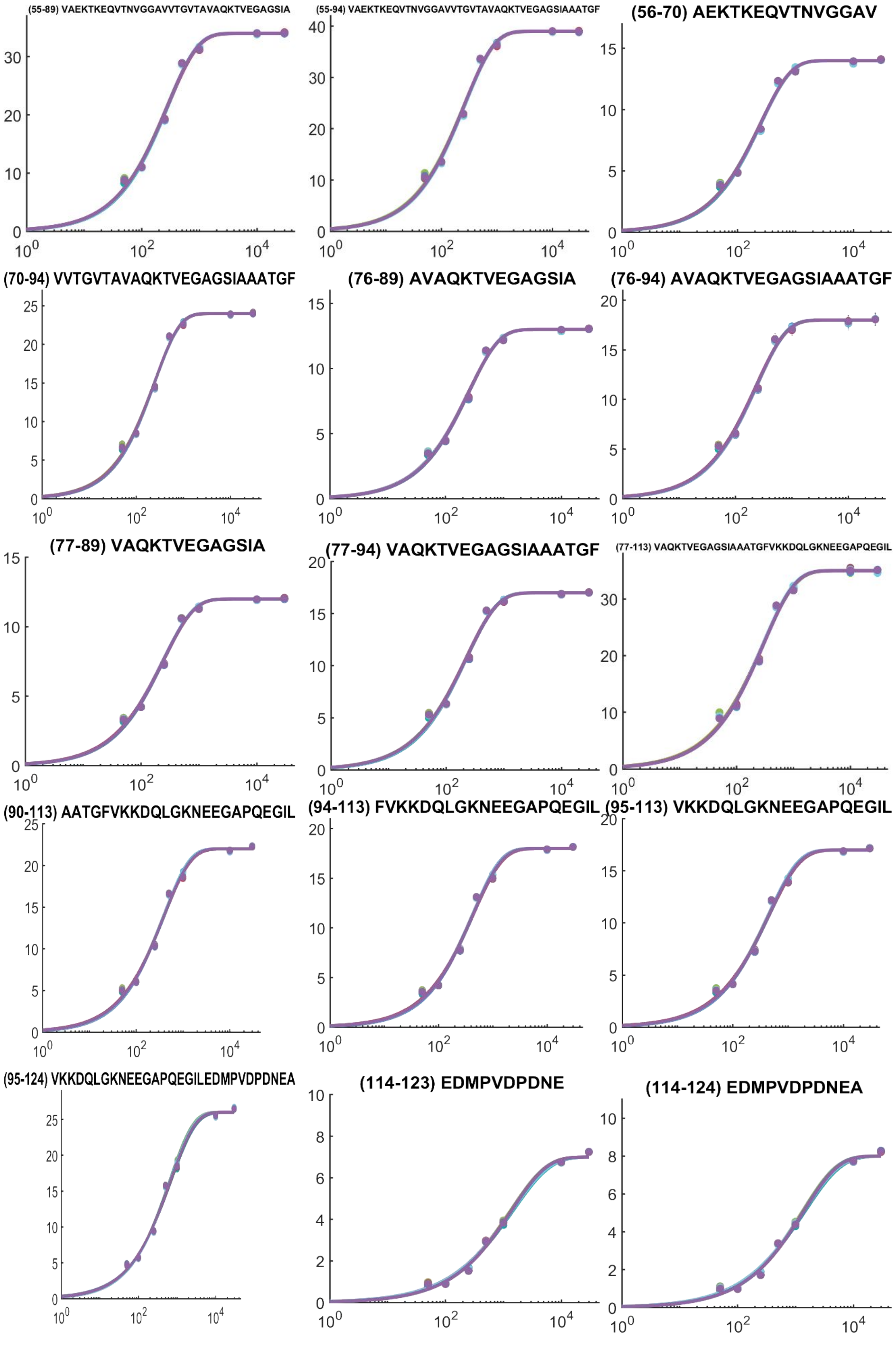

(55-89) VAEKTKEQVTNVGGAVVTGVTAVAQKTVEGAGSIA
(55-94) VAEKTKEQVTNVGGAVVTGVTAVAQKTVEGAGSIAAATGF
(56-70) AEKTKEQVTNVGGAV
(70-94) VVTGVTAVAQKTVEGAGSIAAATGF
(76-89) AVAQKTVEGAGSIA
(76-94) AVAQKTVEGAGSIAAATGF
(77-89) VAQKTVEGAGSIA
(77-94) VAQKTVEGAGSIAAATGF
(77-113) VAQKTVEGAGSIAAATGFVKKDQLGKNEEGAPQEGIL
(90-113) AATGFVKKDQLGKNEEGAPQEGIL
(94-113) FVKKDQLGKNEEGAPQEGIL
(95-113) VKKDQLGKNEEGAPQEGIL
(95-124) VKKDQLGKNEEGAPQEGILEDMPVDPDNEA
(114-123) EDMPVDPDNE
(114-124) EDMPVDPDNEA

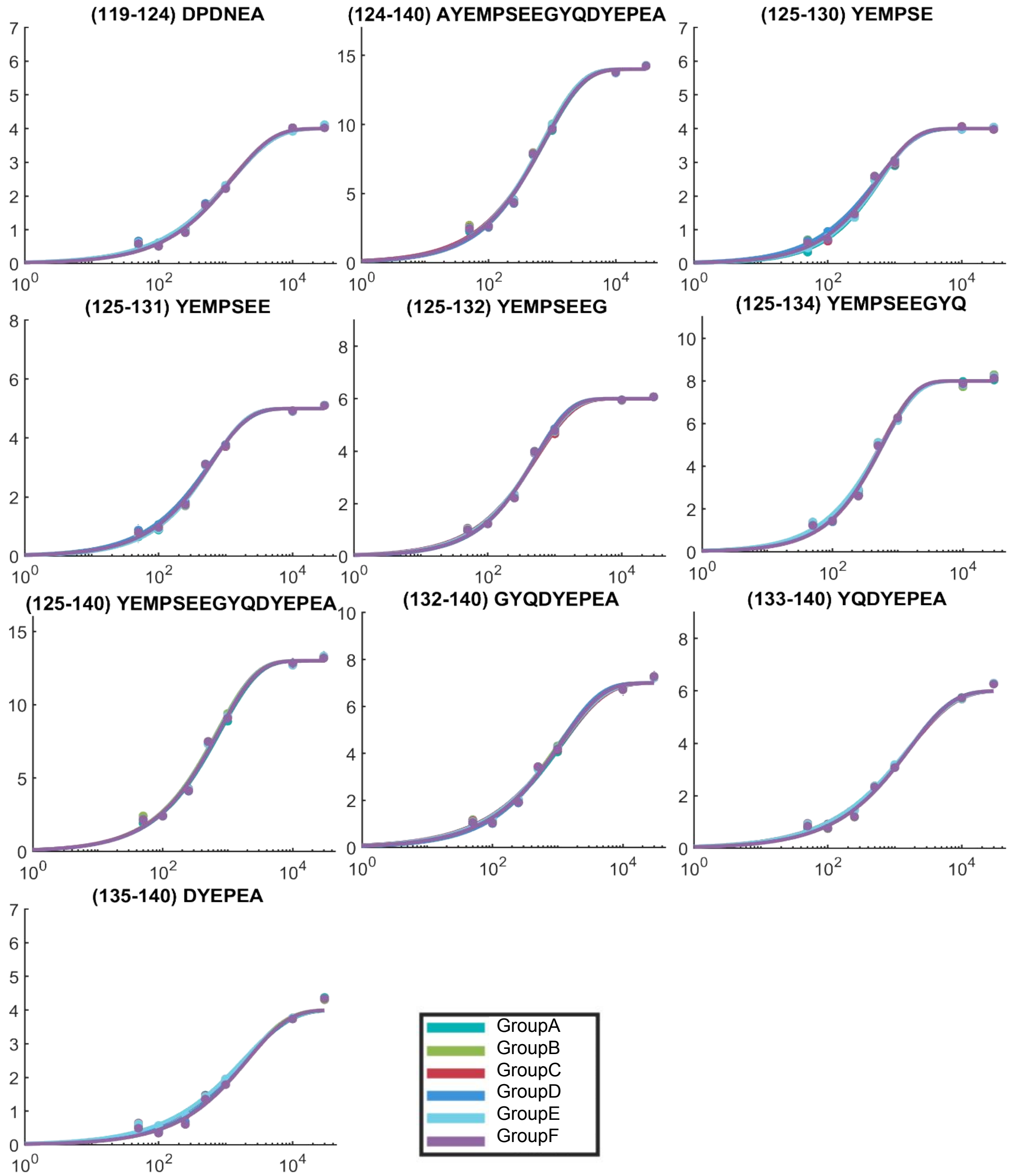

Figure S9: Deuterium uptake plots for the aSyn null experiment.

Table S2: Fitted parameters for the uncorrected and corrected states of bradykinin, and the empirical adjustment factor applied to each fitted parameter.

States	Uncorrected fitting parameters		Corrected fitting parameters		Empirical adjustment factor	
	k	*β*	*k*	*β*	*k*	*β*
Buffer 1	0.0062	0.5247	0.0062	0.5247	1	1
Buffer 2	0.0053	0.5651	0.0062	0.5247	1.179	0.9284
Buffer 3	0.0036	0.6616	0.0062	0.5247	1.722	0.7931
Buffer 4	2.49×10^{-5}	0.5749	0.0062	0.5247	250.1	0.9126

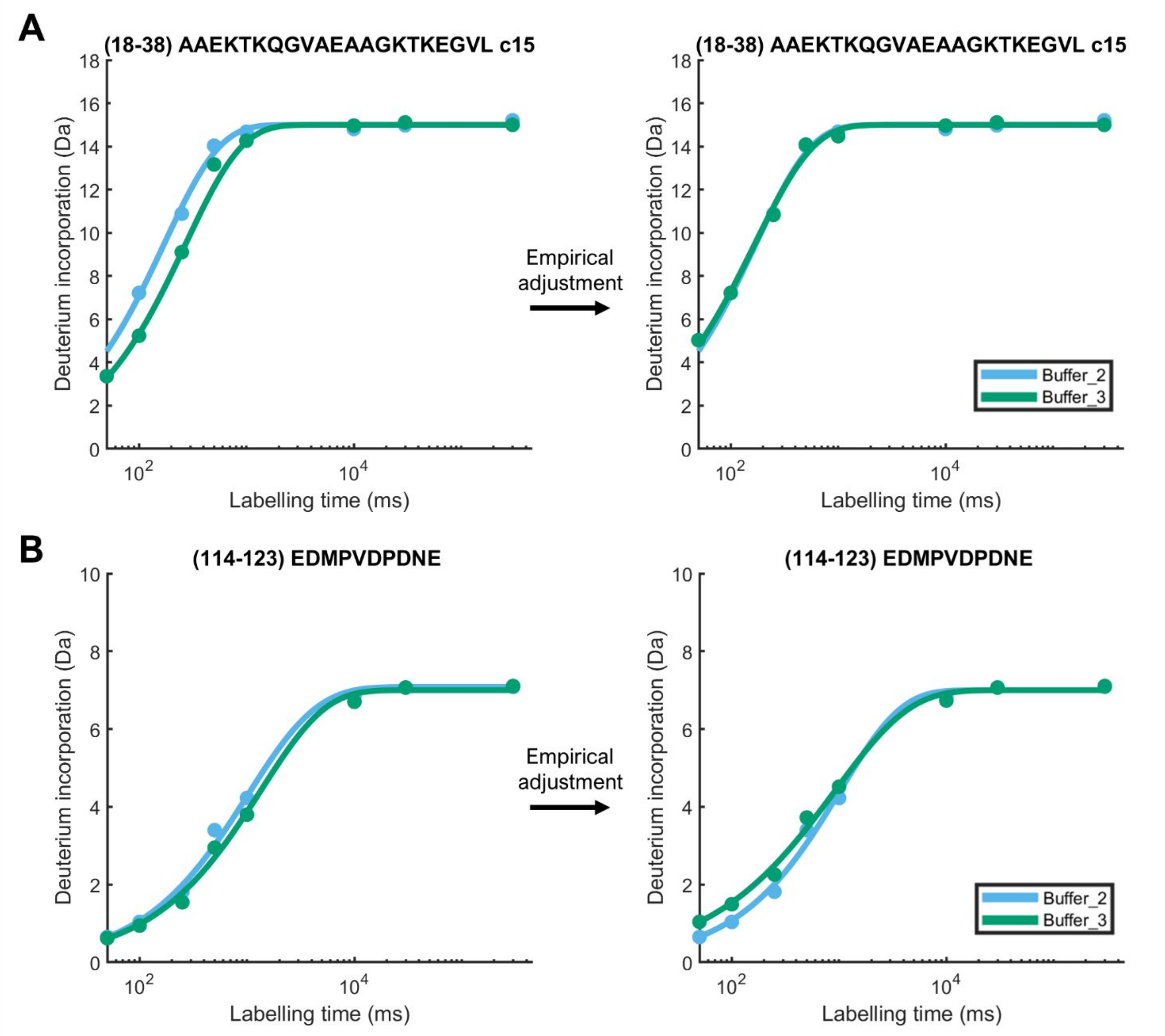

Figure S10: Application of bradykinin-derived empirical adjustment onto aSyn peptide and fragments. **(A)** Fragment 18-38 c15 is completely corrected for when the bradykinin-derived

empirical adjustment is applied, showing that differences between Buffer_2 and Buffer_3 on the left uptake plot are purely due to the intrinsic exchange effects. **(B)** Peptide 114-123 does not fully correct upon empirical adjustment, showing that the differences are due to protein conformational changes.

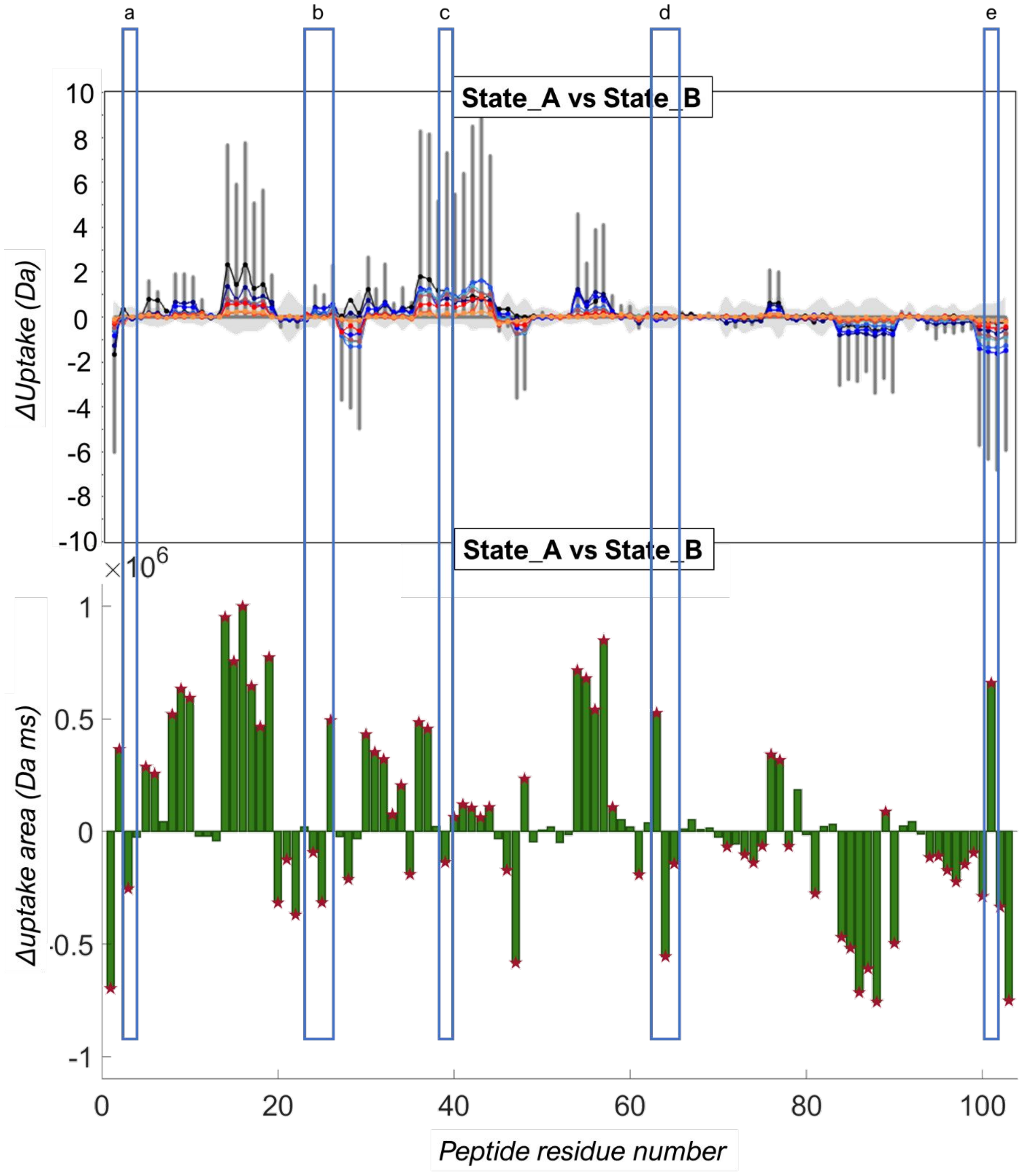

Figure S11: DynamX and `HDfleX` result in distinct difference plots per peptide residue for the comparison of glycogen phosphorylase State_A and State_B. *Top*: the difference 'butterfly' plot output by DynamX shows the difference in the sum of deuterium incorporated over all timepoints up to 300 s between the two states and includes no hybrid significance analysis. *Bottom*: the difference plot output by `HDfleX` shows the difference in uptake area up to 300 s and includes hybrid significance analysis. Drastically different regions between the two comparisons are encased in a blue box, and labelled a-e.
